# Supplementary material for: Characterization of Two Gonadal Genes, zar1 and wt1b, in Hermaphroditic Fish Asian Seabass (Lates calcarifer)
Source: Animals (Basel). 2024 Feb 3;14(3):508. doi: 10.3390/ani14030508 (PMC10854929; doi:10.3390/ani14030508)
Supplement: Supplementary file 1 [file animals-14-00508-s001.zip › animals-2762924-supplementary.pdf]

A

```
1     CGGTTGGTTGAACAAAATGGCAACGTATGGTGAGGAGCCAGTCGACAGCTATTTCTATTCATCTTACAACCCTTACATGGGCAGATATCCCCGAGCTAAAGACGCGGGGTGGAAATATAA
1                      M  A  T  Y  G  E  E  P  V  D  S  Y  F  Y  S  S  Y  N  P  Y  M  G  R  Y  P  R  A  K  D  A  G  W  K  Y  K
121   AAGTTATCTCTCCCACTACGGGGACACTGATGCCTTTAACAACCAGCAGCGCGCTCAGCTCAAGTCCATCTTATCTCAAATCAACCCCAAACTCACCCCGAGGCTCAGGAAGGCAAACAC
36     S  Y  L  S  H  Y  G  D  T  D  A  F  N  N  Q  Q  R  A  Q  L  K  S  I  L  S  Q  I  N  P  K  L  T  P  R  L  R  K  A  N  T
241   CAAAGATGTGGCTGTGCAGGTCAACCCGAAGAGGGACGCCTCGGTACAGTGCTCCATCGGCCCGCGGACGCTCCTGTCCATGAAGAGGGACTTCCGGCGCAAAAGGAGGCAGGAGCCCGA
76     K  D  V  A  V  Q  V  N  P  K  R  D  A  S  V  Q  C  S  I  G  P  R  T  L  L  S  M  K  R  D  F  R  R  K  R  R  Q  E  P  E
361   GACACCCGGCGGCAGCGTCAGCCTCAGTGCGGCGGTCGGTGTGCGTTACCCCCGCACCCTCGCCGTGTACTCGCCTATCGCCTACAGAAGCGTCACCACCTTCCTGGTCGATGACAACAA
116    T  P  G  G  S  V  S  L  S  A  A  V  G  V  R  Y  P  R  T  L  A  V  Y  S  P  I  A  Y  R  S  V  T  T  F  L  V  D  D  N  N
481   CAACAATAAGGAAGCCCCCTGTGGTGAGGCGAAGCCCGCAGAGCCGCCCAGCGACCCGCCCGAGTCTGCGGGGAAAAGCGACGGGAAGAAGAGCGAATATACCCAAGCTGCTGAGGGTGA
156    N  N  K  E  A  P  C  G  E  A  K  P  A  E  P  P  S  D  P  P  E  S  A  G  K  S  D  G  K  K  S  E  Y  T  Q  A  A  E  G  E
601   GAAGGCCGCTAAACAACGAGACCAGCGCGAGAAACTGAAGTCCAAACAGGCGAAGAGCGAAGATGCGCAGCTAACTACAGAGGGGTCAAAGGTCAAGGCGCGTGTGCGTTTCCAGTTTCT
196    K  A  A  K  Q  R  D  Q  R  E  K  L  K  S  K  Q  A  K  S  E  D  A  Q  L  T  T  E  G  S  K  V  K  A  R  V  R  F  Q  F  L
721   GGAACAGAAGTACGGATATTATCACTGCAGAGAATGCAATCTACGATGGGAGAGTGCCTATGTGTGGTGCGTTCAGGGCACCAACAAGGTTTACTTCAAGCAGTACTGTAGAAAATGCCA
236    E  Q  K  Y  G  Y  Y  H  C  R  E  C  N  L  R  W  E  S  A  Y  V  W  C  V  Q  G  T  N  K  V  Y  F  K  Q  Y  C  R  K  C  Q
841   AAAGGACTTCAACCCATATCGTGTTGAGGACATCACATGCCATACTTGCAACAAAGCACGTTGTTCCTGTGCAATAACACAACGCCACGTCGACCCTAAACGGCCCCACAGACAGGACTT
276    K  D  F  N  P  Y  R  V  E  D  I  T  C  H  T  C  N  K  A  R  C  S  C  A  I  T  Q  R  H  V  D  P  K  R  P  H  R  Q  D  L
961   GTGTGGCAGATGCAAAGGCAAGCGGCTCTCCTGCGACAGCACTTTCAGCTTCAAATACATCATCTGAGGAGCACCTCCACTGAACTTTGCCTCCAATACATCAGTAAACGTCCTGTTGTG
316    C  G  R  C  K  G  K  R  L  S  C  D  S  T  F  S  F  K  Y  I  I  *
1081  GAGGGGGCATATTTACAGATGCAATCATTGCATCTTGGATGTAAAAGATATATATTTGTTACTTTATTTCATGCCTGTTTTGTGTTGATGAAATTGATGGTGAGAACCGCAC
```

B

```
1                                                                                                     ACTGTCTCAAACCGCCTTCATCA
24    ATGCTGACTGAGCCATATGGAGCCATGTCCATGGGGTCAGATGTTCGAGATCTGACCCTCCTAACTCCGGCGCCGCCCGTGCCATCCTTGCCAGGAGCTGGCGGCGGCTGTGGAATGTCC
1      M  L  T  E  P  Y  G  A  M  S  M  G  S  D  V  R  D  L  T  L  L  T  P  A  P  P  V  P  S  L  P  G  A  G  G  G  C  G  M  S
144   GTTGGCAGCGGCCAGTGGACCCAACTGCTGGACCTCCACCCTGGCTCTCCCTACAGCTCCCTGCCCTCCCACCACTCCCTCATCAAGCAGGAACCAGGTTGGGGGACCACCGACTCCATG
40     V  G  S  G  Q  W  T  Q  L  L  D  L  H  P  G  S  P  Y  S  S  L  P  S  H  H  S  L  I  K  Q  E  P  G  W  G  T  T  D  S  M
264   GAGGACCCTCACTGCGGACTCGGGGCCTTCACAGTGCACTTCTCAGGCCAGTTTACGGGGTCAGGCCCCTGTCGGGTCGGGGCCTTTGGAGAACCAACTACAGGTCAACCCAGGGTGTTT
80     E  D  P  H  C  G  L  G  A  F  T  V  H  F  S  G  Q  F  T  G  S  G  P  C  R  V  G  A  F  G  E  P  T  T  G  Q  P  R  V  F
384   CCCAATGGAACCTACCTGCCCTGTGTGGACAGCCCCCCTGCATCCAGGAACCAGGGCTACGGAGCGGTGGGTTTAGACAGCAACCCCAGTTACGGCCACACTCCGTCTCACCACGCCCCT
120    P  N  G  T  Y  L  P  C  V  D  S  P  P  A  S  R  N  Q  G  Y  G  A  V  G  L  D  S  N  P  S  Y  G  H  T  P  S  H  H  A  P
504   CAGCTCTCCAGCCTGTCCTTCAAACATGAGGACACCCTGTCACCGCCGAGCAACATAGTTGACCAGCAGTACCCGGCTCCTCCTCCCATGTTTGGCTGCCACAATCCTTCAGAATCCTGT
160    Q  L  S  S  L  S  F  K  H  E  D  T  L  S  P  P  S  N  I  V  D  Q  Q  Y  P  A  P  P  P  M  F  G  C  H  N  P  S  E  S  C
624   CCAAGCAGCCAAGCTCTGCTGCTGAGAAACTACAACAGTGATAACGTATACCAAATGGCGTCTCAGCTGGAGTGTGTAACATGGAACCAAATGAACACCCTGGCGTCTTCCATGAAGAGC
200    P  S  S  Q  A  L  L  L  R  N  Y  N  S  D  N  V  Y  Q  M  A  S  Q  L  E  C  V  T  W  N  Q  M  N  T  L  A  S  S  M  K  S
744   AGTGGCCATACAACCAGCTATGACAGTGACCCCACAGTCCCACCTCCACCCATGCTCGTCAGTGCGCAGTACCACATACATACACACGGTGTCTTCAGGGGACTACAGGATGTCAGACGG
240    S  G  H  T  T  S  Y  D  S  D  P  T  V  P  P  P  P  M  L  V  S  A  Q  Y  H  I  H  T  H  G  V  F  R  G  L  Q  D  V  R  R
864   GTACCTGGCATTGCTCCACCAGTTGTGAAGTCCTCAGAGGCCAACGAGAAGCGTCCGTTTGTATGTGCTTATCCTGGCTGCAGCAAGAGATACTTCAAACTGTCACATCTGCAGATGCAT
280    V  P  G  I  A  P  P  V  V  K  S  S  E  A  N  E  K  R  P  F  V  C  A  Y  P  G  C  S  K  R  Y  F  K  L  S  H  L  Q  M  H
984   GGCCGCAAACACACAGGAGAGAAGCCCTACCAGTGTGATTTCACAGACTGCGGCCGCAGATTCTCTCGCTCAGACCAGTTAAAGAGGCACCAGCGCAGACACACAGGAGTGAAGCCCTTT
320    G  R  K  H  T  G  E  K  P  Y  Q  C  D  F  T  D  C  G  R  R  F  S  R  S  D  Q  L  K  R  H  Q  R  R  H  T  G  V  K  P  F
1104  CAGTGCGAGACGTGTCAGAGAAAGTTTTCACGGTCAGATCATCTTAAGACGCACACTCGGACTCATACAGGTGAGAAGCCCTTTACCTGCCGCTGGTCCAACTGTCAGAAGAAGTTTGCC
360    Q  C  E  T  C  Q  R  K  F  S  R  S  D  H  L  K  T  H  T  R  T  H  T  G  E  K  P  F  T  C  R  W  S  N  C  Q  K  K  F  A
1224  CGCTCTGACGAGCTGGTGCGCCACCACAGCATGCACCAGAGGAACCTGACCAAGCTGCAGCCTGCCATCTGAGCAGCGAGCGAGAGGAGGAGGAGGACGAAGAGGAAGGTGACAATATGT
400    R  S  D  E  L  V  R  H  H  S  M  H  Q  R  N  L  T  K  L  Q  P  A  I  *
1344  TGTGCCAGCTAGTGAAGAGAGGGATGCTTGGAGCCTGGTCAGCAGTTGCGGTGCCTTGTCAGACTCGCTGAAGTGGTGAGCACAGCTGAGGCCTCCCCTCAGCCAGGCAGACAACGTCAG
1454  GAGAGAACACACTCGCTGGTGCTCCACCGATTTAAGATGCACCTCAACTAGTGCTTGCAATCACTAGT
```

Figure S1 The cDNA sequences and predicted peptides of *Lczar1* (A) and *Lcwt1b* (B)

Grey shadow: Zinc-binding domain in (A), and Zn-finger domain in (B). Stop codon is labeled with an asterisk (*).
